# Empowering Quality of Life Monitoring and Self‐Management in Bipolar Disorder: Pilot Evaluation of the PolarUs App

**DOI:** 10.1111/bdi.70096

**Published:** 2026-03-05

**Authors:** Erin E. Michalak, Emma E. Morton, Denny Meyer, Greg Murray, Heather L. O′Brien, Steven J. Barnes

**Affiliations:** ^1^ Department of Psychiatry University of British Columbia Vancouver Canada; ^2^ School of Psychological Sciences Monash University Clayton Australia; ^3^ Centre for Mental Health and Brain Sciences Swinburne University of Technology Melbourne Australia; ^4^ School of Information University of British Columbia Vancouver Canada; ^5^ Department of Psychology, Undergraduate Program in Neuroscience University of British Columbia Vancouver Canada

**Keywords:** bipolar disorder, eHealth, engagement, mHealth, mobile health, self‐management

## Abstract

**Objectives:**

Smartphone apps facilitate the dissemination of resources to help people with bipolar disorder (BD) implement self‐management strategies. However, current apps do not address all treatment outcomes valued by people with BD, nor are they designed with scalability in mind. This study evaluated the feasibility and preliminary efficacy of an alpha version of the co‐designed PolarUs app, a self‐guided intervention developed to support quality of life (QoL) self‐monitoring and self‐management in BD.

**Methods:**

North American residents with a confirmed diagnosis of BD used the iOS PolarUs app for 12 weeks. To assess feasibility, adherence rates were assessed, operationalized by completion of weekly in‐app Brief Quality of Life in BD (QoL.BD) scores. Linear mixed modeling was used to test the hypothesis that QoL (primary outcome) would improve over the intervention period and explore secondary outcomes (i.e., mood symptoms, self‐efficacy, subjective recovery, self‐compassion). Hierarchical cluster analysis was used to investigate associations between app adherence and primary outcomes.

**Results:**

In 170 participants (70% women, mean age 39 years SD = 12.1) there was a steady decline in app adherence over the intervention period, with 37% of participants completing their final weekly assessment. However, significant improvements were observed overall for QoL, mood symptoms, and self‐compassion. Four distinct app adherence clusters were observed, displaying varying relationships with baseline QoL and trajectories of QoL improvement.

**Conclusions:**

Preliminary adherence and efficacy data for the PolarUs app are positive and demonstrate how the inclusion of lived experience perspectives in app development supports intervention acceptability and impact.

## 1 Introduction

The potentially serious effects of bipolar disorder (BD) on health and wellbeing are well‐recognized: beyond the direct experience of mood symptoms, many individuals with BD experience difficulties with interpersonal relationships [1], physical health [2], cognition [3], and occupational functioning [4]. Distressing psychological impacts include stigma, disruption to sense of self, and loss of hope [5]. Optimal treatment of BD therefore involves not only symptom management but also attention to quality of life (QoL), a construct that encompasses health, functioning, well‐being, and satisfaction with various life domains [6, 7]. People with BD consider QoL improvements to be as important as symptom reduction when evaluating treatments [8]. As correlations between mood stability and QoL are typically small to moderate [9], interventions *specifically* targeting QoL are warranted.

Positively, research demonstrates that individuals with BD can live well by employing various strategies to manage symptoms and improve QoL [10, 11]. Self‐management is now recognized as a central component of BD care in major treatment guidelines [12, 13]. Digital mental health interventions (DMHIs), particularly smartphone apps, are one means of improving access to and reach of BD‐related self‐management information and support [14], and have potential advantages over in‐person care. For example, DMHIs are accessible at a time and place convenient to the user, can be used discreetly, and can provide in‐the‐moment interventions and feedback. However, there is widespread concern about the quality, safety, and efficacy of commercial apps [15]. A review of publicly available apps for BD found that one third lacked privacy policies, only one was evidence‐based, and several included harmful, stigmatizing, or misleading content [16]. Individuals with BD report using apps designed for the general public or unipolar depression for mood and sleep self‐management [17]. This is potentially problematic, as such apps may suggest self‐management strategies that risk triggering or exacerbating manic symptoms [18].

In a recent review of app‐based interventions for BD, Anmella and colleagues identified 13 evaluations (randomized control trials, RCTs, and observational studies) of 8 interventions [19]. Most interventions offered self‐monitoring, cognitive behavior therapy, or psychoeducation, alone or in combination. While these strategies fall under the umbrella of self‐management, they may not address broader aspects of QoL and functioning, which people with BD have described as a limitation of current symptom‐focused care [20]. Two apps identified in the review targeted self‐management skills and addressed aspects of wellness beyond symptoms; however one was designed for serious mental illnesses and not BD specifically. The other app, Life Goals, contained information on BD treatment, symptoms, and lifestyle factors. However, user satisfaction with this app was low, with less than half of the participants endorsing it as helpful in managing their health or supporting wellness goals.

To address limitations in the availability of apps targeting the needs and preferences of people with BD, a new DMHI, the PolarUs app, was co‐designed by researchers, healthcare providers, and people with lived experience of BD to support QoL self‐monitoring and self‐management. Core to the app is the Quality of Life in Bipolar Disorder (QoL.BD), a valid and reliable BD‐specific QoL scale [6, 9]: app users self‐monitor their QoL using this scale, and the PolarUs content is specifically designed to address QoL.BD‐relevant life domains measured by this scale. The self‐management content of the PolarUs app was drawn in part from an existing website providing evidence‐informed self‐management strategies for BD [21]. To improve international scalability, the PolarUs app was designed as a fully self‐guided program.

In this paper we report on the quantitative outcomes from a mixed methods pilot evaluation of the PolarUs app. Qualitative and behavioral data findings from the study will be reported in subsequent publications. The study had three quantitative objectives: 1. To assess feasibility of the PolarUs app, with feasibility understood as adherence with the app, measured by number of weekly Brief QoL.BD's completed during the intervention period; 2. To test the hypothesis that QoL (measured using the Brief QoL.BD) would improve across the intervention period. Secondary efficacy outcomes included mood symptoms, self‐efficacy in illness management, subjective recovery, and self‐compassion; 3. To investigate the association between adherence with the PolarUs app and primary and secondary outcomes. Patterns of adherence were assessed using a novel cluster analysis, which was then used as a predictor of primary outcomes.

## 2 Methods

### 2.1 Study Design Overview and Co‐Design Methods

This study was a non‐randomized pilot evaluation of the alpha version of a new app (PolarUs) developed to support QoL self‐monitoring and self‐management in people with BD [22]. The target sample size was 150 adults with a confirmed diagnosis of BD residing in North America. The app evaluation period was 12 weeks. A mixed methods research approach [23], was embedded in the study design; qualitative interviews were conducted with a subsample of participants purposefully selected on the basis of quantitatively assessed varied engagement patterns. These data will be reported elsewhere.

The PolarUs app was developed using co‐design methods by the Collaborative Research Team to Study Psychosocial Issues in Bipolar Disorder (CREST.BD), which specializes in the inclusion of people with BD in the cycle of research and knowledge exchange [10]. Lived experiences of BD were fully integrated into both the development of the app and the design and execution of the evaluation study. For example, some study co‐investigators and staff live with BD. App development and study design and delivery were guided over a two‐year period by a 7‐member advisory group. All advisory group members had lived experience of BD and additional expertise such as user interface design, graphic design, and plain language writing. Development of features and content for the app was also informed by the results of a large (*n* = 919) international survey of use of and attitudes toward apps among people with BD [24] and a survey of healthcare providers [25].

### 2.2 PolarUs App

At the heart of the PolarUs app is the QoL.BD scale, which provides both the organizing framework for the app and serves as the primary assessment tool for evaluating QoL outcomes. The QoL.BD is a valid and reliable BD‐specific QoL scale, available in both Full (56‐item) and Brief (12‐item) form [6]. As described below, the Full QoL.BD assesses wellbeing across 14 QoL domains. The PolarUs app also incorporates the evidence, resources, and tools provided in CREST.BD's Bipolar Wellness Centre [26], wherein BD self‐management content is organized by 17 areas (recognizing that the QoL.BD physical health domain encompasses diet, exercise, substance use and sexual health); app content is also organized according to these 17 areas. The PolarUs app content, however, represents a major expansion beyond that provided in the Bipolar Wellness Centre. Nested within the 17 QoL areas are 99 concrete self‐management strategies and 350 associated resources to support users with implementing these strategies (e.g., worksheets, videos, webinars, articles), produced by CREST.BD or curated from international evidence‐informed sources. A bank of 1479 discrete daily affirmations were also incorporated in the app.

#### 2.2.1 User Flow

**FIGURE 1:**
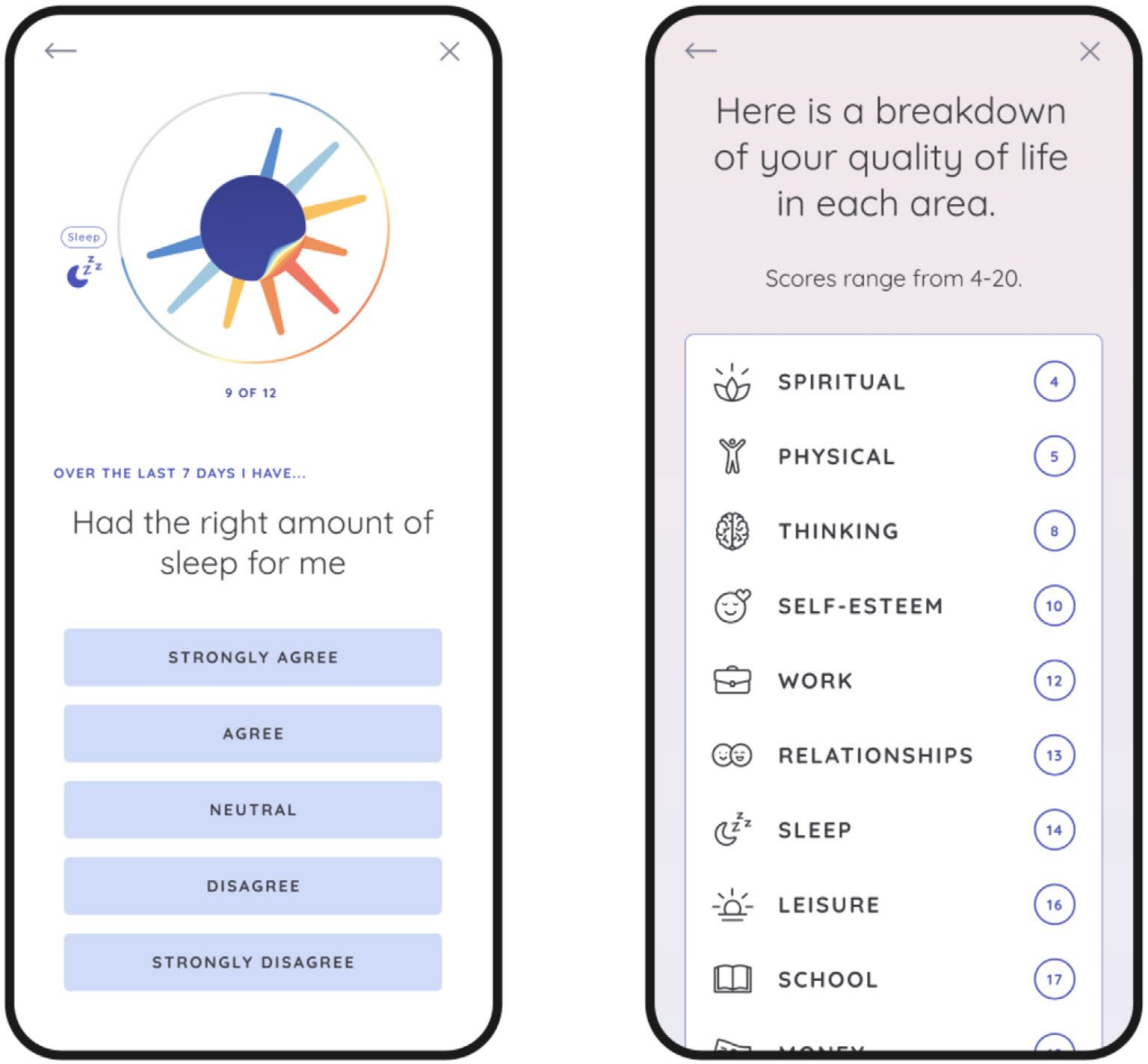
Left: Illustration of the presentation of an item from the QoL.BD. Right: Illustration of a Brief QoL.BD score summary screen.

**FIGURE 2:**
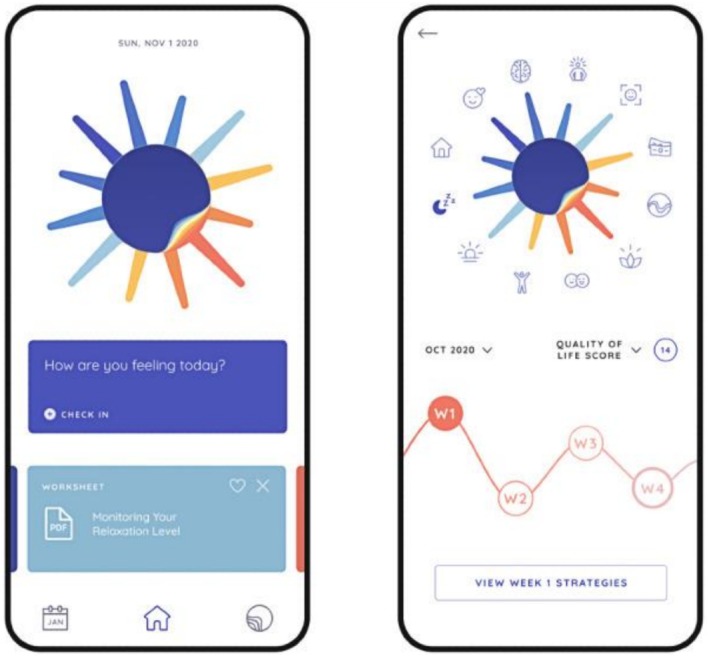
Left: Illustration of a daily mood check‐in prompt and selected resource reminder. Right: QoL.BD history page.

**FIGURE 3:**
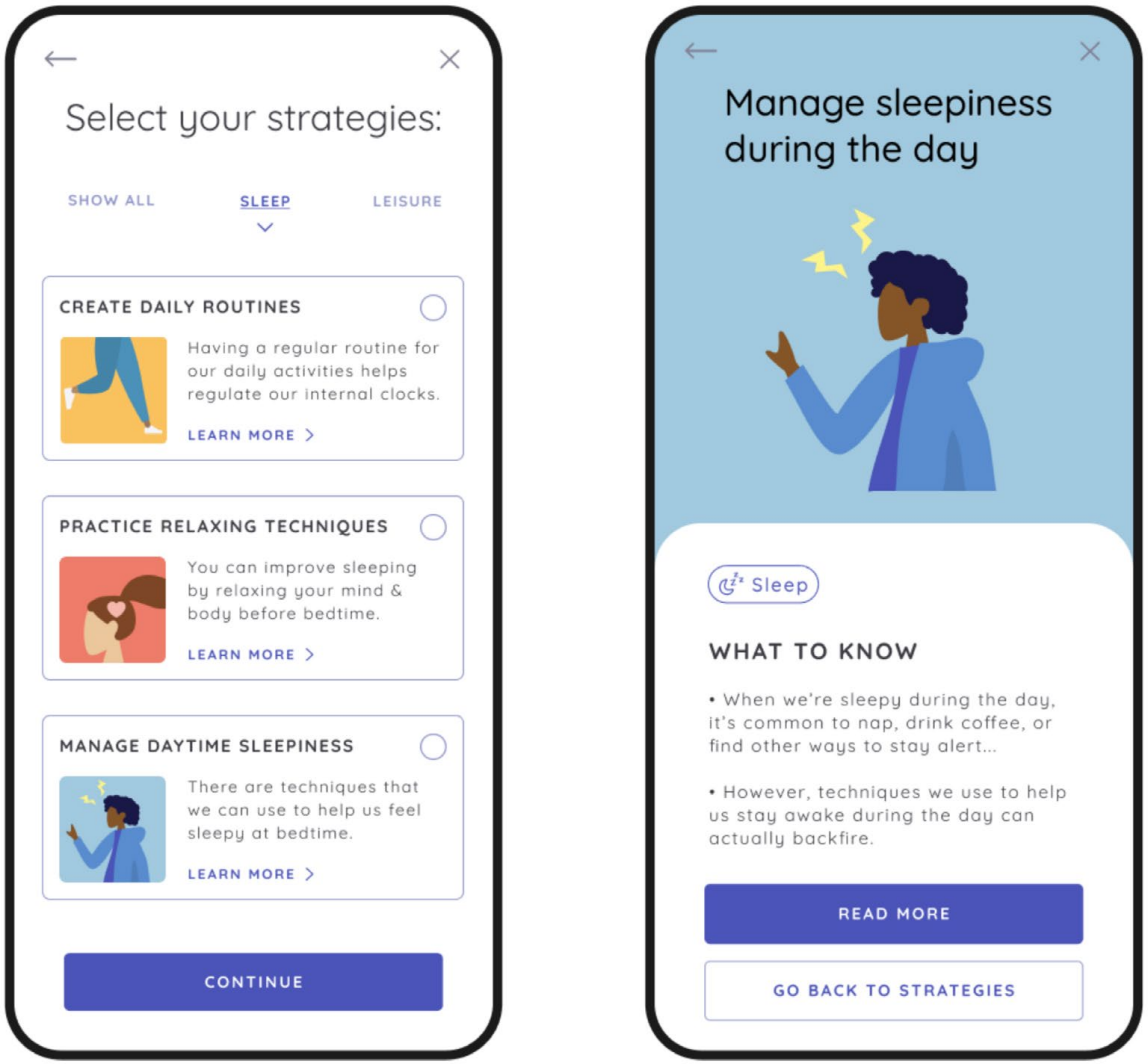
Left: Illustration of strategy selection screen (sleep domain). Right: Illustration of psychoeducation content for selected strategy (manage daytime sleepiness).

In‐app onboarding includes viewing of brief videos explaining the purpose and design of the app and a description of QoL and self‐management in BD. Users are instructed to complete the full QoL.BD at baseline and are provided with feedback on scores across QoL domains. They are then able to select up to three QoL domains to focus on, after which they can select up to four relevant self‐management strategies to implement over the coming month. Once users have selected their self‐management strategies, they are provided with a list of resources related to each strategy to review at their leisure. Users are prompted to complete the Brief QoL.BD on a weekly basis. After 1 month of app use, users are instructed to complete the Full QoL.BD again, at which point they can continue to focus on the same QoL areas or select alternative domains (and strategies) to focus on. The app also incorporates a function for daily monitoring of mood and sleep. Examples of user flow through the app are provided in (Figures 1, 2, 3). Fulsome description of design features of the PolarUs app are provided elsewhere [22].

#### 2.2.2 Technical Specifications

**FIGURE 4:**
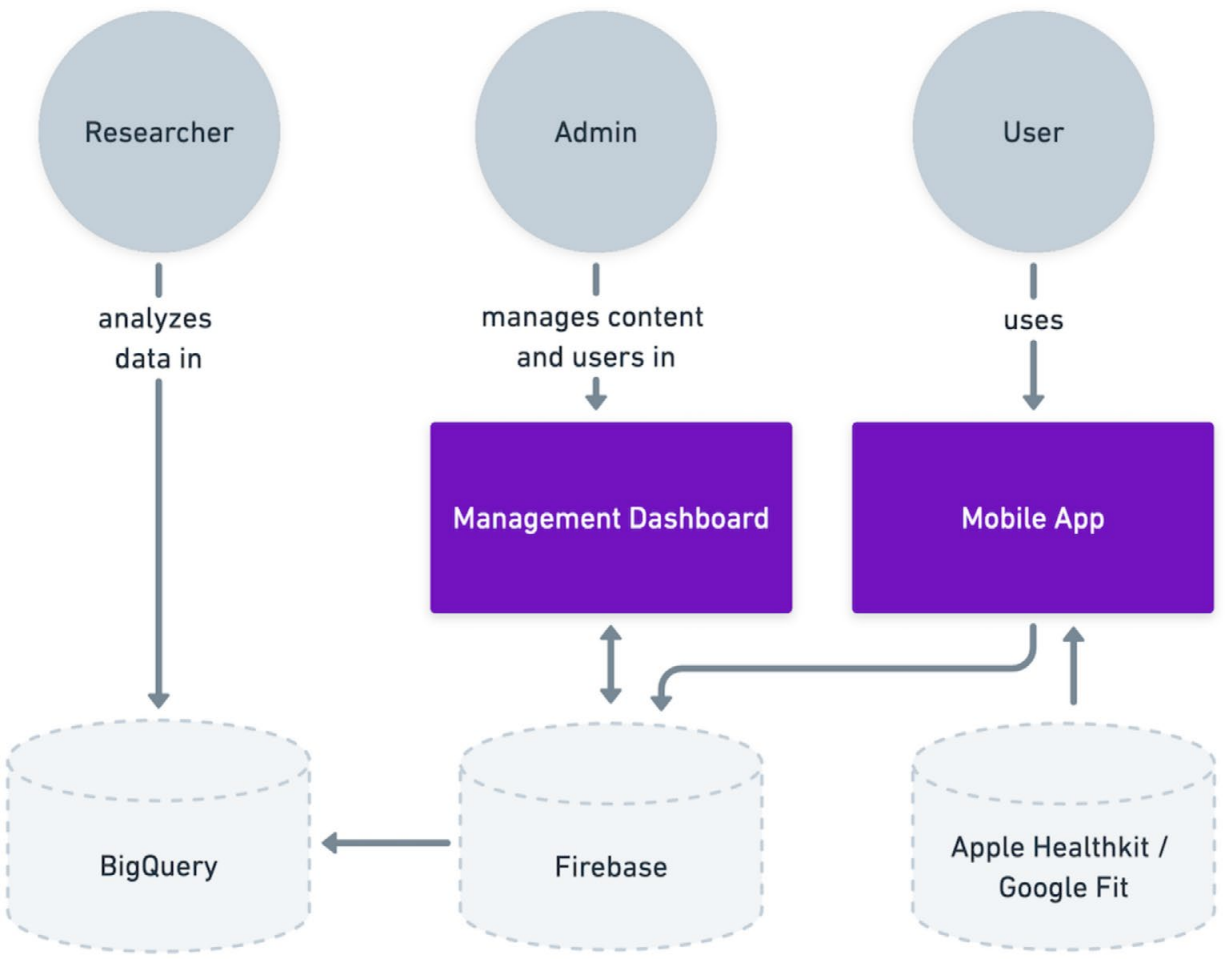
PolarUs app architecture.

The alpha version of the PolarUs app operated on the iOS smartphone platform. It was built using the open‐source Maslo platform (maslo.ai; https://github.com/HeyMaslo), which incorporates several technologies. For its frontend, the Maslo platform uses React Native (https://reactnative.dev), Three.js (https://threejs.org), and Javascript/Typescript. For its backend, the Maslo platform uses Firebase. In addition to using the Maslo platform, the PolarUs app used Neo4J (https://neo4j.com) as its graph database. App architecture is described in (Figure 4).

### 2.3 Participant Recruitment and Inclusion/Exclusion Criteria

Recruitment notices were circulated via the CREST.BD newsletter and shared with partner organizations across North America. The study was promoted on CREST.BD social media platforms (i.e., Facebook, Twitter/X, and Instagram), website (https://crestbd.ca), a project‐specific landing page (https://polarus.app) and via study blogs. Recruitment of the target sample of *n* = 150 participants was reached in 5 months. Examination of the demographics of this initial sample indicated sub‐optimal diversity; purposeful sampling was therefore used to recruit an additional *n* = 20 participants prioritizing inclusion of people who identified as trans or non‐binary, or being from diverse ethnocultural backgrounds. Community partners such as the Depression and Bipolar Support Alliance (DBSA) supported this targeted recruitment by highlighting the study to DBSA peer support group facilitators for Black individuals with mood disorders.

For inclusion, participants were required to be residents of North America, aged > 18 years, with a primary DSM Fourth Edition, Text Revision [27], diagnosis of BD type 1, BD type 2, or BD not otherwise specified as assessed by the Mini‐International Neuropsychiatric Interview (MINI) version 7.0 [28]. Minimally restrictive exclusion criteria were set to aid generalizability; those currently experiencing psychosis or with active suicidal ideation as assessed by diagnostic interview were excluded and provided with resource lists. Participants were also required to be iOS smartphone users, agree to install the app, agree to receive app notifications, and have sufficient understanding of written and spoken English to provide informed consent and engage with the app. Ethics approval was obtained from the University of British Columbia Behavioral Research Ethics Board office (H21‐02042).

### 2.4 Data Collection and Assessment Scales

The MINI was administered by research assistants at baseline via Zoom to confirm diagnostic eligibility. This structured baseline interview was also used to record sociodemographic variables and assess exclusion criteria. Clinician‐rated and self‐report scales were completed at baseline and at monthly intervals during the 12‐week study period. All self‐report scales were administered via a secure, encrypted web‐based survey platform (UBC Qualtrics) with data stored on Canadian servers. All data collected by the PolarUs app was transmitted using end‐to‐end encryption to a secure database on a Canadian server. Research assistants monitored the completion of Qualtrics questionnaires on a regular basis and prompted participants to facilitate completion of the online forms when support was required. At the end of the intervention period, a subset of consenting participants was purposefully selected for invitation to participate in qualitative interviews (data not included in this report). Assessment procedures and study instruments are described below and in (Table 1).

**TABLE 1:** Summary of Subjective and Objective Data Collected.

Data type	Outcomes	Scale	Delivery method (frequency)
Subjective	Condition‐specific QoL^a^ General QoL Chronic Disease Self‐Efficacy Mood Personal recovery Self‐compassion	Full QoL.BD^b^ Brief QoL.BD WHOQOL‐BREF^c^ Stanford's Chronic Disease Self‐Efficacy: “Manage Disease in General” subscale Positive and Negative Affect Schedule Bipolar Recovery Questionnaire Self‐Compassion Scale‐Short Form	In‐app (monthly) In‐app (weekly) Qualtrics (monthly) Qualtrics (monthly) Qualtrics (monthly) Qualtrics (monthly) Qualtrics (monthly)
Objective	Diagnosis Depressive symptoms Manic symptoms	MINI^d^ Montgomery‐Asberg Depression Rating Scale Young Mania Rating Scale	Zoom (baseline) Zoom (monthly) Zoom (monthly)

^a^ QoL: quality of life.

^b^ QoL.BD: Quality of Life in Bipolar Disorder.

^c^ WHOQOL‐BREF: Brief World Health Organization Quality of Life.

^d^ MINI: Mini‐International Neuropsychiatric Interview.

#### 2.4.1 Brief QoL.BD: Primary Outcome Measure

The QoL.BD is the first and (to‐date) only instrument developed to specifically assess QoL in terms of the life areas prioritized by people with BD [6]. The Full 56‐item QoL.BD assesses 12 core (physical, sleep, mood, cognition, leisure, social, spirituality, finance, household, self‐esteem, independence, and identity) and 2 optional (work and study) life areas, each containing 4 self‐report Likert scale items (1: strongly disagree to 5: strongly agree). An overall score (range 48–240) can be calculated by summing the responses to the 48 items of the 12 core domains, with higher scores representing greater life satisfaction. The Brief QoL.BD contains 12 items representing the core domains (overall score range 12–60). During initial field testing, both versions of the QoL.BD had excellent internal reliability (Cronbach α > 0.8), and the Brief QoL.BD demonstrated a higher sensitivity to changes in clinician‐rated symptoms of depression than generic QoL measures [6]. The QoL.BD has been used in international clinical trials, with sensitivity to treatment effects demonstrated [9]. Construct validity of the Brief and full QoL.BD has been demonstrated through associations with symptoms of mania and depression, generic QoL instruments, and functioning [6, 9]. A web‐based adaptation of the full instrument, the QoL Tool, has been psychometrically validated [29]. The Brief QoL.BD served as the primary outcome measure in this study; study feasibility was operationalized as the number of in‐app weekly completions of the Brief QoL.BD.

#### 2.4.2 Mood Rating Scales

The 10‐item Montgomery‐Asberg Depression Rating Scale (MADRS) [30], was administered using a structured interview guide [31]; MADRS scores range from 0 to 60, with higher scores indicating greater severity of depressive symptoms. Symptoms of mania were assessed using the Young Mania Rating Scale (YMRS) [22], an 11‐item scale with scores ranging from 0 to 60, with higher scores indicating greater severity of manic symptoms.

#### 2.4.3 Self‐Report Scales

The Brief World Health Organization Quality of Life (WHOQOL‐BREF) scale [32] was used to assess BD non‐specific aspects of QoL. Chronic Disease Self‐Efficacy was assessed using the 5‐item Manage Disease in General subscale of Stanford's Chronic Disease Self‐Efficacy Scale (SEMDG) [33]. Self‐reported mood was measured using the Positive and Negative Affect Schedule (PANAS) [34]. The 36‐item Bipolar Recovery Questionnaire was used to measure experiences of personal (versus clinical) recovery in BD [35]. The Self‐Compassion Scale‐Short Form was used to measure self‐compassion [36]. Demographic questions assessed sex and gender and included the MacArthur Scale of Subjective Social Status [37].

#### 2.4.4 Sample Size

To inform sample size, we benchmarked a meta‐analysis of clinical trials of smartphone apps for depressive symptoms, which estimated dropout rates of 25% to 50% [38]. Allowing for a 33% dropout rate, 90% power, an effect size of 0.5 SD, and a nonsphericity correction of 0.7 for QoL.BD scores for each participant, the required sample size for addressing Objective 2 (assessing the impact of the PolarUs app on QoL outcomes) was estimated at 150 participants. This sample size also allowed similar power levels to address secondary outcomes.

#### 2.4.5 Statistical Analyses

Only participants who completed all baseline assessments were included in analyses (*n* = 170). Descriptive statistics for these 170 participants are presented for demographic data and monthly assessments missed (Table 2). Scales were computed for the primary and secondary measures with Cronbach's alpha used to assess their reliability. Only in the case of the Bipolar Recovery scale were there missing items for some months; these values were imputed using the Expectation Maximization method, assuming that data were missing completely at random.

**TABLE 2:** Demographic and Clinical Data.

	Categories	Frequency	Percentage
Gender	Women	119	70.0
	Men	43	25.3
	Other	8	4.7
Country	Canada	72	42.4
	United States	98	57.6
Marital Status	Single	54	31.8
	Committed Relationship	31	18.2
	Married	61	35.9
	Common‐law	5	2.9
	Divorced	16	9.4
	Other/Prefer not to say	3	1.8
Ethnicity	Black	7	4.1
	East Asian	7	4.1
	Latin America	6	3.5
	South Asian	6	3.5
	White	120	70.6
	Other/Multiple	24	14.1
Education	Did not finish school	1	0.6
	High school	16	9.4
	Post‐secondary diploma/certificate/assoc degree	25	14.7
	Undergraduate degree	62	36.5
	Master's degree	44	25.9
	PhD	10	5.9
	Other	12	7.1
Employment	FT	86	50.6
	PT	23	13.5
	Casual	3	1.8
	Unemployed	13	7.6
	Student FT	13	7.6
	Student PT	2	1.2
	Volunteer	4	2.4
	Retired	6	3.5
	Disability pension	20	11.8
MacArthur Scale of Subjective Social Status	1 (Lowest standing)	6	3.5
	2	12	7.1
	3	15	8.8
	4	18	10.6
	5	32	18.8
	6	34	20.0
	7	38	22.4
	8	12	7.1
	9	2	1.2
	10 (Highest standing)	1	0.6
Type of BD	Type 1	158	92.9
	Type 2	6	3.5
	NOS	6	3.5
Currently in treatment	No	14	8.2
	Yes	156	91.8
App usage on smartphone/tablet	More than once per day	158	92.9
	At most once a day	11	6.5
	Never	1	0.6
Average hrs per day using these apps	At most 1.0	24	14.1
	2.0	37	21.8
	3.0	31	18.2
	4.0	23	13.5
	5 or more	43	25.3
	Missing	12	7.1
Use of health and wellbeing apps	No	43	25.3
	Yes	127	74.7
Monthly Assessments not completed	At 4 weeks	21	11.0
	At 8 weeks	21	11.0
	At 12 weeks	45	23.6
	At Follow‐up	46	24.1

To assess **Objective 1** (to assess feasibility of the PolarUs app, with feasibility understood as adherence with the app, operationalized as number of weekly Brief QoL.BD's completed over the intervention period, rather than more study‐centric feasibility outcomes, such as recruitment or enrollment rates) descriptive statistics were presented on adherence rates overall and across the study period. Potential associations between the number of missed assessments and demographic data were examined using a Generalized Linear Model to predict the number of weekly Brief QoL.BD assessments missed, assuming a Binomial distribution but allowing for over‐dispersion.

To assess **Objective 2** (to test the hypothesis that QoL would improve across the intervention period), a linear mixed model was fitted to the data for the primary outcome measure (weekly Brief QoL.BD) assuming a random intercept and slope. In this model we controlled for country of residence as a fixed factor. This was an intention‐to‐treat analysis, carried out without and with the multiple imputation (MI) of missing data, both assuming that data was missing at random. In both analyses the model was fitted using the Full Maximum Likelihood approach.

A similar analysis was also carried out for the monthly secondary measures after first testing for normality and establishing the nature of the correlation structure for these scales across time, using a random intercept model to estimate the intra‐cluster correlation coefficient (ICC). Finally, marginal means were computed for the monthly assessments with effect sizes (Cohen's d) provided for changes over the intervention period.

To assess **Objective 3** (to investigate the association between adherence with the PolarUs app and primary and secondary outcomes) a Hierarchical cluster analysis was conducted with the number of Brief QoL.BD assessments completed in the 4‐to‐5‐week periods between clinical assessments (i.e., adherence data) using Ward's method, with the best results obtained for the Silhouette measure when there were four clusters. The clusters were graphically compared in terms of their adherence over time and their mean Brief QoL.BD scores in each 4‐to‐5‐week period. Demographic comparisons across the clusters showed no significant relationships so these results are not reported here.

## 3 Results

### 3.1 Demographics and Baseline App Use

Participants were 19 to 81 years old (M = 39, SD = 12.1). The majority of participants identified as women (70%), with more participants resident in the United States (57.6%) than Canada (42.4%) as indicated in (Table 2). The majority described their ethnicity as white (70.6%) and most were either single (31.8%) or married (35.9%). Many had an undergraduate degree (36.5%) or a postgraduate degree (31.8%) and were in full time employment (50.6%). Scores on the MacArthur Scale of Subjective Social Status [37], tended to be low to moderate (with 61% rating themselves as 5–7). Participants had a diagnosis of BD I (92.9%), BD II (3.5%) or BD NOS (3.5%). The majority (91.8%) were currently receiving treatment for their BD. The majority used smartphone apps for at least 2 h a day (78.8%), and 74.7% were using health or wellness apps.

### 3.2 Objective 1: Feasibility

#### 3.2.1 Missed Assessments

In total 144 participants (84.71%) had some missing Brief QoL.BD data with a total of 781 weeks (35.34%) with missing values. There was a steady decline in completions of 6.7 assessments per week on average over the 13‐week period, with 37% of participants completing their final week 13 assessment. Only country of residence showed a significant relationship with the number of weekly Brief QoL.BD assessments missed (Z = 2.175, *p* = 0.030). On average the number of weekly assessments missed was 33.2% lower for Canada than the United States. In the ensuing analyses we therefore controlled for country of residence in order to address any bias which could be attributed to missing data.

### 3.3 Objective 2: Primary and Secondary Outcomes

#### 3.3.1 Evaluation of Scales

All scales had good internal reliability (Cronbach alpha > 0.80) and indicated an exchangeable correlation structure over time, with a pairwise correlation equal to the ICC as given in (Table 3). In most instances, the scales suggested a normal distribution; however, this was not the case for the Negative Affect scale of the PANAS and the SEMDG, which had positive and negative skews, respectively. Transformations were used to correct for the skewness in these scales.

**TABLE 3:** Linear Mixed Model Random Coefficient Model adjusted for Country of Residence without and with Multiple Imputation (MI). Significant Results Bolded.

Scale	MI	Cronbach alpha	ICC	Mean intercept (I)	Mean trend (T)				Corre‐ lation I with T
					Estimate	Lower limit	Upper limit	*p*	
Brief QoL.BD	No	0.883	0.60	40.88	0.181	0.070	0.292	0.**002**	−0.32
Brief QoL.BD	Yes		0.54	41.08	0.200	0.072	0.328	0.**003**	−0.45
Full QoL.BD	No	0.966	0.56	158.53	3.132	1.173	5.091	0.**002**	−0.69
Full QoL.BD	Yes		0.51	159.89	3.254	0.930	5.577	0.**007**	−0.67
MADRS	No	0.890	0.50	11.78	−1.059	−1.618	−0.499	**< 0.001**	−0.73
MADRS	Yes		0.47	11.50	−0.950	−1.517	−0.382	0.**001**	−0.67
YMRS	No	0.790	0.47	5.98	−0.613	−0.931	−0.296	**< 0.001**	−0.86
YMRS	Yes		0.42	5.85	−0.598	−0.947	−0.249	**< 0.001**	−0.86
WHOQOL	No	0.847	0.72	86.70	1.191	0.598	1.784	**< 0.001**	−0.47
WHOQOL	Yes		0.69	86.85	1.289	0.653	1.925	**< 0.001**	−0.44
SCSSF	No	0.888	0.80	31.42	0.819	0.490	1.148	**< 0.001**	−0.45
SCSSF	Yes		0.78	31.41	0.807	0.410	1.204	**< 0.001**	−0.44
BRQ	No	0.915	0.67	2539.4	20.82	2.252	39.37	0.**028**	−0.63
BRQ	Yes		0.58	2539.8	25.619	7.498	43.74	0.**006**	−0.84
SEMDG^1^	No	0.876	0.70	1312.6	23.65	−1.524	48.82	0.065	−0.47
SEMDG^1^	Yes		0.66	1308.4	25.081	−2.077	52.24	0.070	−0.39
PANASPos	No	0.920	0.44	30.13	−0.335	−0.843	0.173	0.194	−0.80
PANASPos	Yes		0.41	30.11	−0.275	−0.832	0.283	0.331	−0.77
PANASNeg^2^	No	0.880	0.52	3.105	−0.020	−0.041	0.002	0.070	−0.63
PANASNeg^2^	Yes		0.43	3.110	−0.027	−0.058	0.005	0.092	−0.58

Abbreviations: BRQ, Bipolar Recovery Questionnaire; ICC, Intra‐cluster Correlation Coefficient; MADRS, Montgomery‐Asberg Depression Rating Scale; MI, multiple imputation; PANASPos/Neg, Positive and Negative Affect Schedule; SCSSF, Self‐Compassion Scale‐Short Form; SEMDG, Stanford's Chronic Disease Self‐Efficacy “Manage Disease in General” subscale; WHOQOL, WHOQOL‐BREF; YMRS, Young Mania Rating Scale.

^1^ Square transformation to correct for skewness.

^2^ Logarithmic transformation to correct for skewness.

#### 3.3.2 Analysis for Primary Outcome Measure: Brief QoL.BD

**FIGURE 5:**
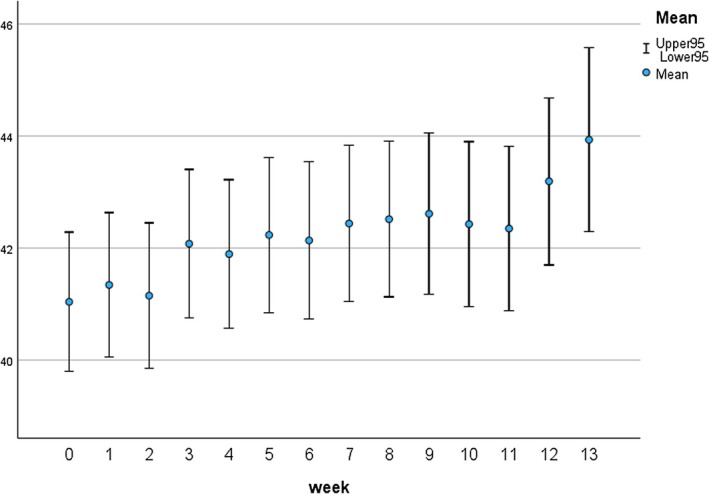
Marginal Means for the Weekly Brief QoL.BD Measures with 95% Confidence Intervals.

An initial random intercept model indicated that 60% of the variation in the Brief QoL.BD could be attributed to participant differences, also indicating an estimated correlation of 0.60 between the results in any 2 weeks. Next, a model with random intercept and random trend was fitted, showing a significant intercept (B = 40.88, 95% CI: 39.48–42.28), t (179) = 57.7, *p* < 0.001 and a significant slope (B = 0.181, 95% CI: 0.070–0.292, t (121) = 3.224, *p* = 0.002). The correlation between the estimated intercepts and slopes was −0.32 across participants, suggesting a faster improvement for participants with lower QoL at baseline. There was a significant improvement in model fit for the model with random intercept and trend over the random intercept model (χ^2^(1) = 111.7, *p* < 0.001), and there was no significance for the Country effect in this model (F (1,163) = 0.195, *p* = 0.659), suggesting that there was no significant bias in the results which could be attributed to country of residence (Figure 5), provides a plot of the marginal means with 95% confidence intervals, with Cohen's d indicating a relatively small improvement over the 13 weeks (d = 0.21). The results in (Figure 5) are based on the original data, without MI. Tests for a fixed or random quadratic trend produced no significant result (*p* = 0.994 and *p* = 0.902, respectively).

#### 3.3.3 Linear Trend Analysis for All Secondary Measures

Table 3 shows the results of the Linear Mixed Model analyses without and with MI of missing values. Trend is measured in terms of assessment number. Results were similar without and with MI. The percentage of variation that can be attributed to participant differences varied between 41% and 80% and the correlation between the intercept and trend coefficient varied between −0.32 and −0.86, suggesting greater improvements when baseline results were poorer. Significant improvements over time are observed for the Full QoL.BD, the MADRS, YMRS, WHOQOL and SCSSF, with a marginally significant improvement for the BRQ. No significant trend was observed for the SEMDG, the PANAS Positive or PANAS Negative.

Marginal means are provided in (Table 4) for each of the secondary measures, validating the trends observed in (Table 3). Cohen's d effect sizes suggest relatively small improvements over the 13 week period.

**TABLE 4:** Marginal Means for Each Monthly Assessment, Adjusted for Country of Residence and Allowing for a Random Intercept (Significant results bolded).

Scale	Marginal means for monthly assessments				Overall significance					
	Ass1	Ass2	Ass3	Ass4	% change	F	df1	df2	*p*	Cohen d
MADRS	11.36	9.67	8.76	8.33	**26.7**	5.60	3	413	**< 0.001**	−0.30
YMRS	5.14	4.10	3.66	3.32	**35.4**	6.09	3	399	**< 0.001**	−0.31
Full QoL.BD	159.9	166.3	168.5	168.5	**5.4**	4.12	3	411	0.**003**	0.24
WHOQOL	87.64	88.92	89.87	91.19	**4.1**	6.02	3	399	**< 0.001**	0.26
SCSSF	33.12	33.99	35.51	35.27	**6.5**	11.59	3	388	**< 0.001**	0.23
SEMDG	36.67	37.11	37.34	37.66	2.7	1.43	3	397	0.235	0.12
PANASPos	29.44	29.09	29.45	28.13	4.4	1.18	3	411	0.318	−0.15
PANASNeg	20.56	20.35	19.58	19.54	5.0	1.43	3	411	0.235	−0.12
BRQ	2532	2536	2567	2590	2.3	1.74	3	407	0.157	0.13

*Note:* Means for SEMDG and PANAS Negative have been back‐transformed to their original measurement scales.

Abbreviations: BRQ, Bipolar Recovery Questionnaire; MADRS, Montgomery‐Asberg Depression Rating Scale; PANASPos/Neg, Positive and Negative Affect Schedule; SCSSF, Self‐Compassion Scale‐Short Form; SEMDG, Stanford's Chronic Disease Self‐Efficacy “Manage Disease in General” subscale; WHOQOL, WHOQOL‐BREF; YMRS, Young Mania Rating Scale.

### 3.4 Objective 3: Associations Between Adherence and Outcomes

**FIGURE 6:**
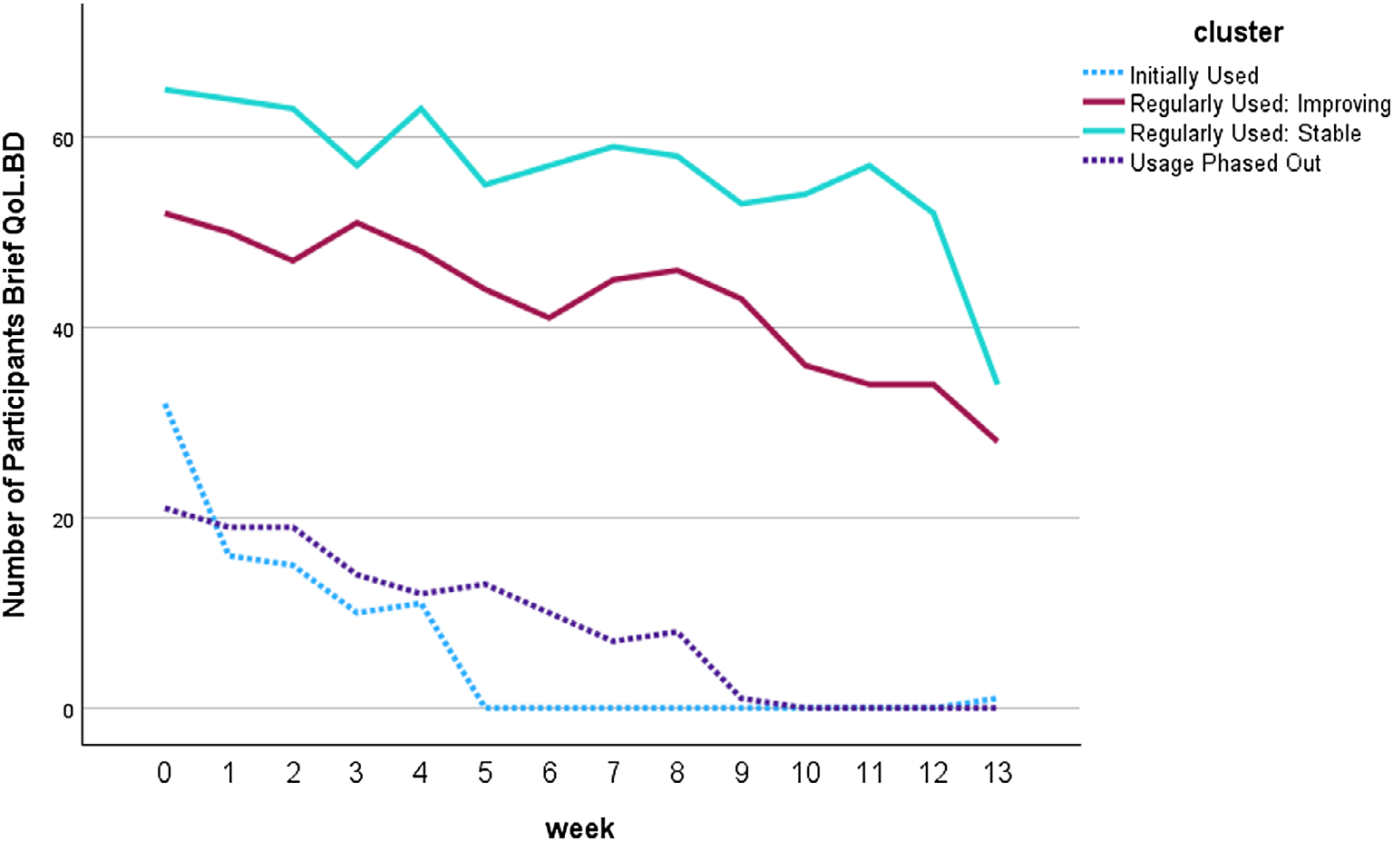
Completion of Brief QoL.BD over Time.

**FIGURE 7:**
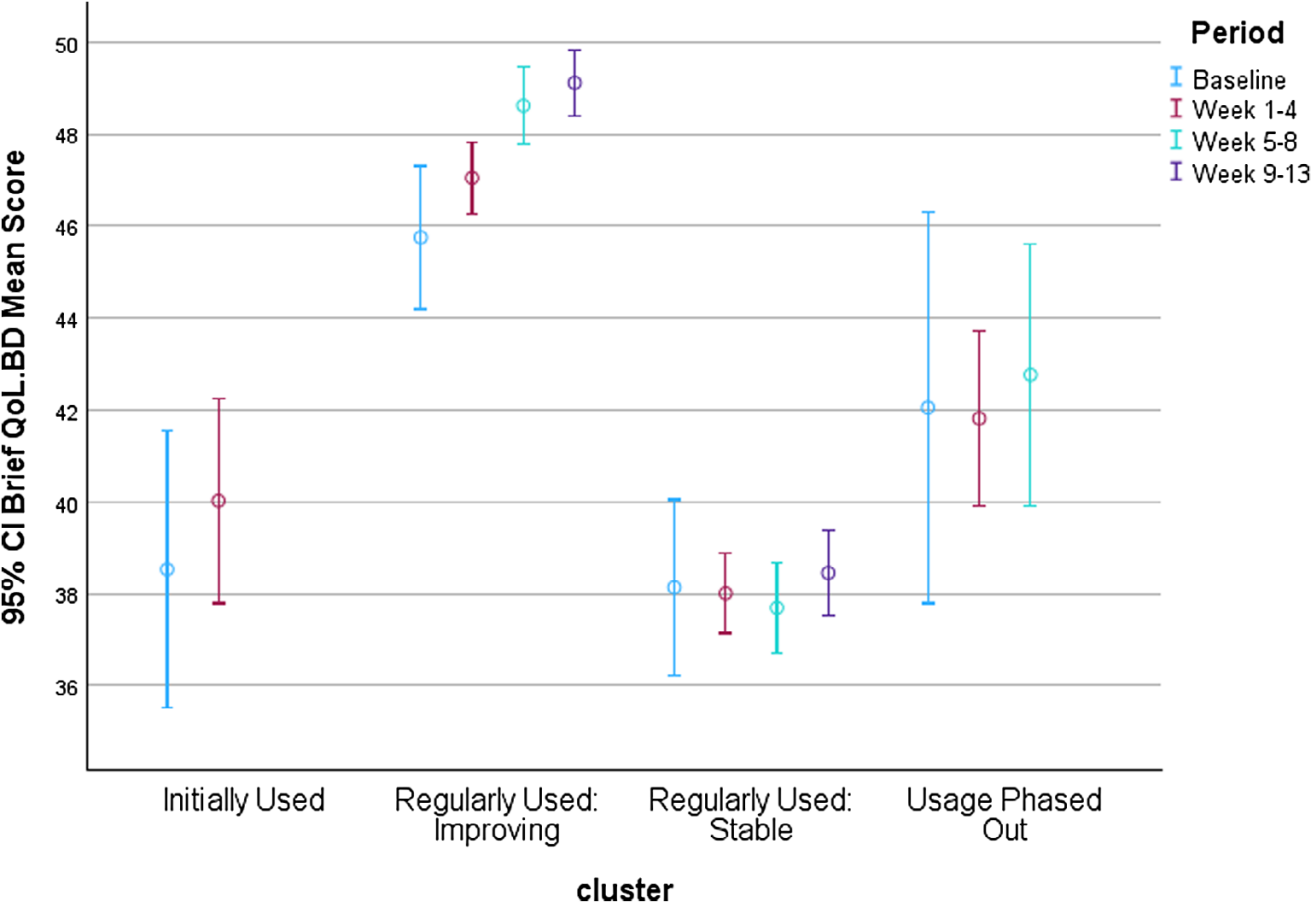
Variation in Brief QoL.BD Mean Scores Across Engagement Clusters.

Four participant clusters were identified using the number of Brief QoL.BD assessment completions in each 4‐ to 5 week period. As illustrated in (Figures 6 and 7) these participant clusters differed in the number of Brief QoL.BD assessments completed in each period and the changes in mean Brief QoL.BD scores observed. Cluster 1 (Initially Used: *n* = 32), cluster 2 (Regularly Used Improving: *n* = 52), and cluster 4 (Usage Phased Out: *n* = 21) showed evidence of improvements in their Brief QoL.BD scores (a steady significant improvement in the case of cluster 2). However, cluster 3 (Regularly Used Stable: *n* = 65) did not show improvement, despite starting from a relatively low base.

## 4 Discussion

This paper described the feasibility and impact of the alpha version of the PolarUs app for BD, as assessed via a single‐arm pilot study. Specific objectives addressed were: (1) To assess feasibility of the PolarUs app, with feasibility understood as adherence with the app, operationalized by completion of weekly in‐app Brief QoL.BD assessments; (2) To test the hypothesis that QoL (measured on the Brief QoL.BD) would improve across the intervention period and to explore secondary efficacy outcomes; and (3) To investigate the association between app adherence and primary and secondary outcomes by way of cluster analysis.

With respect to feasibility (adherence) outcomes, as with many mental health apps, we observed a gradual decline in adherence over the study period. With respect to primary and secondary outcomes, positively, significant improvements were observed in our primary outcome of QoL, as well as assessor‐rated symptoms of mania and depression, and self‐compassion.

Our cluster analysis of adherence rates provided more granular insights on usage patterns and associated impacts, with four distinct clusters displaying varying relationships between adherence, baseline QoL, and trajectories of QoL improvement. A recent systematic review of adherence rates to smartphone apps in BD suggested activity rates between 58%–91% [39], while another review of general DHMI's showed high variability across real‐world and research studies [40, 41]. This may be because the quality of reporting on adherence is poor and unstandardized; for example, downloading the app versus completing all components of the intervention [40]. The current study operationalized adherence as completion of the QoL self‐assessments. This has two implications for our interpretation of adherence. First, our estimate of adherence may not directly map onto engagement, as participants could still access psychoeducation materials and resources *without* completing these assessments. Second, most research studies collect non‐obtrusive behavioral data, e.g., number of logins, pages or app features accessed, time spent [41], that requires no additional effort on the part of app users. A recent systematic review of DMHIs for BD confirmed that use of mental health apps should be as low‐effort as possible by reducing barriers related to accessing and interacting with the app and its content [42]; completing the monthly assessment may have increased some participants' cognitive load resulting in lower completion levels, even as they continued to use and potentially benefit from PolarUs. Indeed, qualitative data explored in detail elsewhere highlighted some barriers to completion of QoL self‐assessments, including negative emotional responses to seeing lower than desired responses to self‐monitoring data [43], and a higher level of perceived effort associated with the longer weekly and monthly QoL self‐assessments as compared to daily check‐in items [43]. Future evaluations of the PolarUs app will therefore incorporate exploratory analyses of behavioral indicators of engagement collected in‐app.

Further, it is difficult to determine the ‘dose’ of DMHIs required for meaningful user impact, and different people may benefit from different ‘doses' [40]. As a result, usage data may offer only part of the picture when it comes to user engagement with DMHIs, and may not account for subjective relevance or utility. In this study, QoL—not user engagement—was the primary outcome variable, and this differs from many commercial health applications. User engagement in DMHI research is often bluntly equated to behavioral metrics of app usage rather than a holistic interpretation of usage and its relationship to positive health outcomes [44, 45]. As noted, QoL is an individualized experience: “individuals with BD locate their QoL in relation to a variety of reference points drawn from past experiences, social context or expectations for the future” [20]. This study allowed participants to select QoL indicators most relevant to their situation and goals, and to select strategies to work toward these goals. Thus, an area for future analysis of this data will be to determine if there are different patterns of engagement for QoL areas based on the self‐report and usage data we collected. A forthcoming qualitative analysis will also explore in greater detail factors that influenced engagement with the app [43]. This qualitative data will complement the present quantitative analysis by exploring salient aspects of engagement from the perspective of participants, including both usage of various app features (self‐monitoring and psychoeducational materials), as well as out of app experiences (e.g., cognitive and emotional responses to app content, attempts to enact self‐management strategies). We expect these analyses will generate hypotheses for potential differing patterns of app usage and benefit reported in this study, and will inform the selection of additional data that should be quantitatively collected and explored in future evaluations.

Low adherence rates with apps may also stem from a dearth of user involvement in app development processes. A prior review found only two of eleven mental health self‐monitoring apps used in research with people with BD could be classified as having a high level of user involvement, defined as users having input across the full spectrum of app development, from conceptualization, to prototype design, to evaluation [45]. Of the two apps identified in this aforementioned review that were developed with high user involvement, neither were designed for BD. A significant strength of this study lies in the fact that it was conducted within a Community Based Participatory Research framework involving people with lived experience of BD and co‐design methods integrated into the app development, study design and execution, and knowledge exchange phases.

One notable study limitation relates to the generalizability of our findings. Regarding language accessibility, the alpha version of the PolarUs app was only available in English; none of its content was translated nor culturally adapted. Lack of availability of culturally and linguistically appropriate interventions represents a substantial barrier to equitable access to DMHIs [46], with representatives of ethnocultural and linguistic minorities often not included in the development and testing of DMHIs [47]. Many DMHIs are therefore not designed to reflect social and cultural diversity, which can lead to low acceptability, feasibility, and effectiveness of interventions among these populations [47]. This is despite the fact that interest in DMHI appears to be high among ethnic and racial minorities [48], suggesting that failure to provide culturally appropriate interventions undercuts their potential as acceptable treatments in these populations.

Further limiting generalizability of our findings is the likelihood that participants with a specific interest in DMHIs and self‐management of BD self‐selected into the research project. Although we aimed to make the PolarUs app as accessible as possible through community consultation, consideration of accessibility issues specific to serious mental health issues during the design phase [49], and attention to reading level in content writing, it is still likely that individuals with more familiarity with apps and computers will be better able to engage with the intervention and complete all required research tasks (i.e., web‐based questionnaires). Individuals with BD who report using self‐management apps for mood and sleep demonstrate higher levels of digital health literacy [50]; therefore, individuals with less confidence in navigating DMHIs may have been less likely to take up or adhere to PolarUs. Although it was not feasible for us to offer dedicated supports to upskill participants in technological abilities in the context of this study, future research may evaluate whether adjunctive interventions can enhance the feasibility of DMHIs in populations with BD. Our sample was also highly educated and over half of the sample reported being in full‐time employment. Most importantly in terms of limitations is the non‐controlled nature of our pilot data; a fully powered RCT is required to definitively assess the efficacy of the PolarUs app.

Positioning this study within the context of the wider DMHI literature, it is encouraging to see that other DMHIs designed for BD populations are in development and/or evaluation. Notable is the LiveWell [51], self‐management app, designed to reduce relapse risk and improve QoL in BD. Outcomes of a recently published RCT (*n* = 205) did not detect a difference in the primary outcome of time to relapse, but positive effects were observed for depression symptom severity and relational QoL. The LiveWell app has a number of strengths, including a user‐centered development process and integration of content addressing both symptoms and QoL. Inclusion of human support in the intervention through six one‐to‐one motivational interviewing coaching sessions and data sharing with healthcare providers may support effective engagement. However, these roles are also a barrier to scalability, as implementation of LiveWell as designed will require dedicated coaching staff and resources. Furthermore, the planned integration of the app in clinical care means it may not reach individuals who are not utilizing formal services due to their inaccessibility, issues of stigma, or beliefs about treatment.

We look forward to a time when people with BD will have access to a palette of options for evidence‐based DMHIs, allowing for self‐selection of apps according to user preferences, needs and context. The PolarUs app, a DMHI specifically targeting QoL and self‐management in BD designed for scalability (i.e., primarily designed for use as a self‐guided program) may add a valuable option in the DMHI landscape. One perennial problem lies in the fact that research‐led DMHIs are rarely made publicly available [52]; researchers are increasingly encouraged, therefore, to formulate plans for sustainable dissemination. To accelerate public access to the PolarUs app we secured philanthropic support from the Daymark Foundation in Canada to advance development of the English language version of the PolarUs app for full market release on iOS and Android platforms. In parallel with data collection for the study reported here, we underwent a 2 year co‐design and development period to produce a significantly advanced beta version of the PolarUs app and the content contained within it. App advances include substantial improvements to user experience and user interface design and additional self‐monitoring options and graphical feedback. With attention to accessibility and diversity, a comprehensive refresh of PolarUs content was conducted: key messages, audiovisual alternatives, and effort ratings were added and new resources tailored for diverse communities (e.g., ethnocultural/racialized, older adults, LGBTQ2S+, Indigenous communities). A future avenue of research will be to evaluate real‐world patterns of uptake and engagement with the beta app to better understand the feasibility of PolarUs across diverse naturalistic settings, including resource‐constrained environments. Further supporting our goal of ensuring the PolarUs app is feasible across cultures, funding has been obtained to culturally adapt the PolarUs app for ethnoculturally diverse communities and to definitively test the efficacy of the advanced app in improving QoL in diverse people with BD within an RCT design.

The results of the present evaluation of the PolarUs app are encouraging and add to a solid body of literature supporting the potential of self‐management for optimizing health and QoL for people with BD and the relatively nascent literature on the effective DMHIs for the condition. However, not all individuals with BD will be interested in, able to access, or able to use DMHIs. Previous qualitative investigations of how service users with serious mental illnesses viewed DMHIs have flagged concerns that such tools would replace support options delivered by a person [53]. As such, supporting individuals with BD to access evidence‐based self‐management and support through diverse intervention streams is important. Catering to individuals who prioritize human interaction, we are developing a non‐app form of intervention delivery, leveraging the content and resources within the PolarUs app to create a peer‐delivered group psychoeducation program. Similar to DMHIs, peer support has been identified as a means to improve access to and affordability of mental healthcare [54, 55, 56]. Peer support can offer additional benefits for subjective recovery: qualitative data from a previous CREST.BD intervention evaluation highlighted unique benefits for individuals who participated in a face‐to‐face group workshop, including reduced self‐stigma, increased self‐compassion, and self‐efficacy [20, 21]. By developing a variety of scalable and effective app and non‐app self‐management interventions (to be used alone or in combination), we hope to extend the reach of evidence‐based self‐management information to all individuals with BD who would benefit.

## Author Contributions

E.E.M. and S.J.B. conceptualized, designed, and executed the overall study. E.E.M. contributed to the study design and execution and led the qualitative analyses. D.M. led the quantitative analyses. G.M. co‐designed the QoL.BD with E.E.M. H.L.O. contributed to the study design aspects focused on engagement metrics. All authors provided critical revision of the manuscript for important intellectual content. All authors have read and approved the final manuscript.

## Funding

This work was supported by Canadian Institutes of Health Research, 201903.

## Conflicts of Interest

S.J.B., G.M., H.L.O., and D.M. declared no potential conflicts of interest with respect to the research, authorship, and publication of the paper. E.M. has received an honorarium for advising on the development of educational materials for Neurotorium, an online educational platform supported by the Lundbeck Foundation. E.E.M. has received funding to support patient education initiatives from Otsuka‐Lundbeck.

## Data Availability

The data that support the findings of this study are available on request from the corresponding author. The data are not publicly available due to privacy or ethical restrictions.
